# A unique transcriptomic landscape defines African-specific grade group 1 prostate cancer

**DOI:** 10.21203/rs.3.rs-9384143/v1

**Published:** 2026-04-24

**Authors:** Eva Ferlev Jensby, Korawich Uthayopas, Md Mehedi Hasan, Melanie Louw, Shingai B.A. Mutambirwa, Phillip Stricker, Raymond Campbell, Philippe Lamy, Luigi Marchionni, M.S. Riana Bornman, Anthony Papenfuss, Karina D. Sørensen, Vanessa M. Hayes

**Affiliations:** Aarhus University Hospital; The University of Sydney; The University of Sydney; National Health Laboratory Service; Sefako Makgatho Health Sciences University; St Vincent’s Hospital Sydney; Steve Biko Hospital; Aarhus University Hospital; NewYork–Presbyterian Hospital; University of Pretoria; Walter and Eliza Hall Institute of Medical Research; Aarhus University Hospital; The University of Sydney

**Keywords:** African ancestry, transcriptomics, prostate cancer, grade group 1

## Abstract

**Background:**

Prostate cancer (PCa) exhibits significant ancestry-related disparity. While men of African ancestry experience higher overall mortality rates, this difference is most pronounced in Sub-Saharan Africa and for grade group 1 (GG1) disease, alluding to ancestry-specific biology. Despite this health disparity, African-relevant and prostate tumour GG1 inclusive data, specifically transcriptomic data, is lacking. In turn, this raises significant concerns with regards to adopting Eurocentric models to classify and manage assumed indolent disease for African men. The risk - suboptimal treatment decisions.

**Methods:**

Using a single technical and analytical pipeline, we generated total RNA sequencing data from fresh-frozen prostate tissue for 68 Black South African (40 GG1-PCa, 28 non-PCa) and 48 Australian European men (all GG1-PCa), performing ancestry-specific differential gene expression and pathway analysis. Sourcing public data enabled limited African American inclusive The Cancer Genome Atlas cross-validation (13 of 61 GG1-PCa), while Pan Prostate Cancer Group European ancestral data provided for deeper cross-ancestral comparative analyses (106 GG1-PCa, 17 non-PCa).

**Results:**

Identifying 5,652 differentially expressed genes between African and European ancestral GG1 tumours (*p* < 0.05), including top-ranked PCa tumour suppressor genes *DUSP1, JUN, FOS*, and *JUNB* downregulated in African tumours. In turn, six metabolic and six immune-related pathways showed significant African-specific negative enrichment. Concordantly, cell type analysis showed significantly lower immune, stromal, and angiogenesis scores in African over European-derived GG1 tumours. Inclusion of African American GG1 data showed pathway over gene-level ancestry-specific concordance, with significant negative enrichment verification for oxidative phosphorylation, fatty acid metabolism and glycolysis. Compared to and irrespective of PCa status, our African tissues showed a 4.9-fold increase in differential gene expression in PSA-high versus PSA-low tissues. Notably, cell type clustering revealed 29% of PSA-high non-PCa tissues exhibited cancer-like profiles, indicating potential occult disease.

**Conclusions:**

Revealing substantial transcriptomic divergence from European ancestral GG1 tumours, we identify African-specific transcriptomic features that may contribute to outcome disparities in this under-appreciated clinical group. Our study highlights not only a critical shortcoming in providing equitable PCa care for African men, but it also raises major concerns with regards to managing and treating African men using European-developed criteria.

## Introduction

Prostate cancer (PCa) is the second most frequently diagnosed cancer and the fifth leading cause of cancer-related death in men worldwide^1^. However, in most countries across Sub-Saharan Africa, PCa is the leading cause of male-associated cancer death. The disproportionately high mortality rate can be attributed to several factors, including late-stage presentation, limited healthcare infrastructure, and under-resourced screening programmes^2^. Furthermore, delayed presentation leads to an under-representation of low-risk or International Society of Urological Pathology (ISUP) grade group 1 (GG1) PCa in African men^3^. Consequently, little is known regarding the presentation, prevalence, and aetiology of GG1-PCa within the African setting. However, what is emerging is that men of African ancestry experience a 2-fold greater GG1-PCa associated mortality over European ancestral men from the same health care system^4^, prompting the question whether the underlying biology of GG1 disease may differ by ancestry.

Biased towards aggressive disease, tantalising evidence exists that men of African ancestry present with different biological and genomic features, concluding that African ancestral high-risk disease disparity cannot be explained by socioeconomic factors alone^5^. Most notably, and even in the absence of detectable PCa, African men present with elevated PSA levels^6^. More recently, we showed that compared with African American men, southern and east African men diagnosed with PCa were 3-fold more likely to present with PSA levels associated with the National Comprehensive Cancer Network (NCCN) classification for high/very-high-risk PCa (PSA ≥ 20 ng/mL)^7,8^. This highlights the biological heterogeneity within the broad African identifier. Additionally, while testosterone levels are elevated in Black over White Americans, levels are further exacerbated for Black South Africans, with a rapid age-associated decline associated with increased PCa risk^9^. At the genomic level, earlier analyses discovered a higher tumour mutational burden, increased molecular heterogeneity and taxonomy specific to African over non-African PCa patients^10^. In addition, *TMPRSS2-ERG* fusions, a prevalent driver of PCa in European patients, occur less frequently in men of African ancestry^11^, who also exhibit distinct mutational profiles and copy-number alterations compared to Asian and European populations^12^. Although previous studies indicate a significantly different genomic taxonomy in African over European ancestrally derived tumours, studies focused on transcriptomic profiling prostate tumours, including GG1 tumours, from populations across sub-Saharan Africa low-grade are lacking^13^. The consequence - missed or under-appreciated clinically relevant variance, leading to poor decision-making for African patients across the globe.

In this study, we performed total RNA sequencing on prostate tissue from 68 Black South African men (34 GG1, 6 atypical small acinar proliferation (ASAP), 28 non-PCa) and 48 European Australian patients (all GG1-PCa). This unique GG1-focused data resource enabled ancestry-specific differential gene expression (DGE) and pathway analysis. Tumour tissue from African men showed a distinct transcriptional profile compared with European-derived tissue, with negative enrichment of multiple metabolic and immune pathways and an epithelial-dominant tumour microenvironment (TME). Moreover, among African men with high PSA levels, a subset of non-PCa tissues displayed cancer-like characteristics, which may suggest limitations in current clinical practices leading to exacerbated misdiagnosis for African patients. Our findings were validated using The Cancer Genome Atlas (TCGA) African American inclusive (13 of 61 GG1-PCa)^14^ and Pan Prostate Cancer Group (PPCG) European restricted resource (106 GG1-PCa, 17 non-PCa). Collectively, our observed transcriptomic features provide important insights into a largely overlooked PCa diagnosis - African-specific high-risk GG1 disease.

## Methods

### Participant recruitment, presentation, and ethics

Participants comprised 68 South African men of African ancestry and 48 Australian men of European ancestry (Table 1). South African men were recruited at a Southern African Prostate Cancer Study (SAPCS) participating urology clinic, including Dr George Mukhari Academic (n = 49), Steve Biko Academic (n = 11), or Kalafong Hospitals (n = 7) in Pretoria, Gauteng Province, while a single patient was recruited from Tshilidzini Hospital in Vhembe, Limpopo Province. Men were diagnosed through transrectal ultrasound-guided (TRUS) biopsies, with the first sampled core snap-frozen for downstream analysis. Ancestral self-identification was provided for two generations through ethno-linguistic identification, including prefix-omitted Southern Bantu identifiers Ndebele, Pedi, Shangaan, Sotho, Tsonga, Tswana, Venda, Xhosa, and Zulu, while a single patient self-identified as South African Cape Coloured, including both African and non-African ancestral fractions. Of these 68 men, 28 had no detectable PCa, including 20 with benign prostatic hyperplasia (BPH), 34 were diagnosed with GG1-PCa, and six with ASAP. In this study, patients with ASAP were classified within the GG1-PCa group, as ASAP frequently reflects biopsy sampling error with non-inclusion of a cancerous area, and low-grade PCa is commonly detected on repeat biopsy^15^. This is consistent with the use of low-sensitivity TRUS biopsies, which often miss anteriorly placed tumours; a trait common in PCa patients of African ancestry^16,17^. Excluding ASAP samples from the African PCa group did not materially alter the African PCa versus European PCa comparison (Pearson r = 0.96, *p* < 0.001 for log_2_ fold changes), supporting their classification as PCa.

All self-identified European ancestral Australian men were diagnosed with GG1-PCa and elected to undergo radical prostatectomy (RP) at St Vincent’s Hospital in Sydney. A fresh-frozen palpation-guided biopsy core from each RP specimen was provided for this study. All histopathological data were reviewed for the SAPCS by M.L., while Australian data was provided by the Garvan Institute St Vincent’s Prostate Cancer Biobank.

### Public data resources

From the publicly available TCGA_PRAD dataset^14^ (Table 1), we analysed 61 patients whose race was noted as “Black or African-American” (13 GG1-PCa) or “White” (48 GG1-PCa). RNA sequencing and clinical data were publicly available and downloaded from the GDC data portal of the NIH National Cancer Institute, USA. Genes with ≤ 10 reads in more than 13 samples were removed to enhance analytical robustness. DGE analysis between Black and White American was performed using *DESeq2*^18^ (v.1.44.0) on the dds object.

For non-African validation of our African GG1-PCa versus non-PCa results, we obtained batch-corrected total RNA-seq data (106 GG1-PCa, 17 non-PCa) from the PPCG (Table 1). The PPCG consortium processed raw reads with *Cutadapt*^19^ (v.3.4), *Salmon*^20^ (v.1.4.0, GENCODE v38 lifted to GRCh37), and *tximport*^21^ (v.1.18.0), with normalisation and RUVIII-PRPS batch correction^22^ (k = 10) applied to the full dataset before providing GG1-subset data for this analysis.

### RNA purification and total RNA sequencing

All fresh-frozen biopsy or RP samples weighing less than 10 mg were purified using the QIAwave DNA/RNA Mini Kit (Qiagen, Germany) following the manufacturer’s instructions. The purified RNA had a median RIN score of 2.6 and was sequenced with the Illumina Stranded Total RNA prep with Ribo-Zero Plus workflow and sequenced on a NovaSeq 6000 system using an S4 flow cell with 2× 150 bp paired-end reads with an aim of 70 million reads per sample. Following sequencing, all samples were quality-checked (QC) using *FastQC*^23^ (v.0.12.1). Adapters (ACTGTCTCTTATACACATCT) were removed from both read ends using *CutAdapt*^19^ (v.5.1) with quality trimming at Q20 and a minimum length of 20 bp. Transcripts were quantified using *Salmon*^20^ (v.1.10.1) with GENCODE v47 GRCh38 as the reference transcriptome using k-mer size 17, selective alignment mode (--validateMappings), GC bias correction (--gcBias), and automatic library type detection. Transcript-level estimates were aggregated to gene-level counts using *tximport*^21^ (v.1.36.1), based on GENCODE v47 transcript-gene mappings, with raw counts imported for downstream analysis. Gene-level counts were filtered, normalised and variance-stabilised using *DESeq2*^18^ (v.1.44.0), following recommended procedures for *tximport*-processed data to account for gene length.

### Statistics

All analyses were performed in R (v. 4.4.1) using R Studio (v. 2026.01.0.392). Patients in this study were divided into PSA low/high subgroups based on their corresponding ancestry median (13.75 ng/mL African, 4.4 ng/mL European), resulting in 29 African PSA-high, 30 African PSA-low, and 1 African PSA unknown, and 20 European PSA-high, 21 European PSA-low, and 6 European PSA unknown. To evaluate associations between continuous numerical variables and ancestry, cancer status, or PSA groups, we employed the Wilcoxon rank-sum test. Spearman’s rank correlation coefficient was used to quantify associations between transcription factor expression levels and pathway enrichment scores, computed separately for patients of African and European ancestry. Pearson’s product–moment correlation coefficient (Pearson’s ρ) was applied to assess concordance of log_2_ fold changes between this study and TCGA.

DGE analysis was performed using *DESeq2*^18^ (v.1.44.0) directly on the dds object, and *p*-values were adjusted for multiple testing using the Benjamini-Hochberg (BH) approach. Gene set enrichment and overrepresentation analyses (ORA) were performed using the R packages *fgsea*^24^ (v.1.30.0) and *clusterProfiler*^25^ (v.4.12.6), respectively, employing the Hallmark gene sets as well as WP_AR_SIGNALING and WP_AR_NETWORK_IN_ PROSTATE_CANCER gene sets from MSigDB^26^. Gene set variation analysis (GSVA) was performed using the *GSVA*^27^ (v. 1.52.3) package. Heatmaps were generated using the *ComplexHeatmap*^28^ (v. 2.20.0) package, employing Ward’s minimum variance method for hierarchical clustering and Spearman distance metrics.

To assess the cell type composition in each sample, we devised cell type signatures by including the expression of well-known gene markers for basal epithelial cells (*KRT5*, *KRT14*, *TP63*)^29,30^, luminal epithelial cells (*KLK3*, *AR*, *NKX3–1*, *FOLH1*)^30,31^, stromal cells (*ACTA2*, *COL1A1*, *VIM*, *TAGLN*)^32^, immune cells (*CD68*, *CD3E*, *TNF*, *CXCL8*, *IL-6*, *CCL2*)^33,34^ and endothelial cells (*PECAM1*, *VEGFA*, *FLT1*)^35,36^. We calculated the cell type signature score as the mean log_2_ expression of all marker genes for each cell type. In the calculation of immune scores in the TCGA cohort, *CD68* was not included, as its expression did not exceed the quality detection threshold in this dataset.

To estimate tumour content in the African men, we calculated a tumour content estimation defined as:

$$\text{Tumour}\;\text{burden}\;\text{score}=(\text{z\_scaled}\left(\frac{\text{AMACR}+\text{PCA3}+\text{HOXC}4+\text{HOXC}6+\text{FOLH}1}{5}\right)-\text{z\_scaled}\left(\frac{\text{TP6}3+\text{KRT}5+\text{KRT}14}{3}\right)$$

The resulting score is a tumour content estimation score, where higher values indicate more tumour-like expression. The markers chosen are widely recognised PCa markers (Additional File 2: Fig. S1a)^29,30,37–40^. The score significantly distinguished African PCa samples from non-PCa samples (*p* = 0.0052; Additional File 2: Fig. S1b). The signature was divided into four groups representing: Very high, high, moderate, and low tumour content based on the quartiles in the African men with PCa.

## Results

### Clinicopathological presentation for ancestrally assigned GG1-PCa patients

Irrespective of country of origin (68 South Africa, 48 Australia), all fresh-frozen prostate tissue cores underwent sample processing, total RNA sequencing, and associated analysis, using a single technical and analytical pipeline (see Methods). South African men, self-identifying ethno-linguistically as African or Southern Bantu were recruited at a participating SAPCS urology clinic (Table 1). Recruited at diagnosis, 34 (48.6%) presented with histopathologically confirmed PCa defined as GG1, six (8.8%) presented with ASAP (suspicious lesions grouped with PCa, see Methods), while 28 (41.2%) had no detectable PCa (non-PCa), 20 with BPH and 14 with prostatitis. Conversely, the 48 Australian men self-identified as European and were recruited at the time of elective surgery for pathologically confirmed GG1-PCa from St Vincent’s Hospital in Sydney, Australia. Notably, our southern African men presented on average 8.2 years later, with significantly elevated PSA levels over our European cases (median 11.8 vs 4.4 ng/mL, *p* = 5.6^−10^, Wilcoxon rank-sum test). Concurring with previous population-matched observations^6,8^, PSA levels for our non-PCa African controls mirrored levels observed for cases (median 14.4 ng/mL).

### Ancestry-derived PCa gene expression and pathway enrichment

Samples with an effective library size less than 15 million (n = 3; 1 PCa, 2 non-PCA) and/or less than 10,000 expressed genes (read count ≥ 10; n = 9; 7 PCa, 2 non-PCa), were removed (Additional File 2: Fig. S2 and S3a-b). As three samples failed both criteria, a total of 9 samples were removed. To increase analytical robustness, genes expressed in less than 26 samples were removed, with 26 representing the smallest biological group present in the data (26 non-PCa, 53,237 genes removed). The final dataset comprised 107 samples (34 African GG1-PCa, 26 African non-PCa, 47 European GG1-PCa; Additional File 1: Table S1; Additional File: Fig. S4) and included 25,040 genes (Additional File 2: Fig. S3c). Principal component analysis (PCA) of the top 1,000 genes with the highest median absolute deviation in the entire dataset revealed no clear separation between GG1-PCa and non-PCa or between median defined PSA-high and PSA-low tissues (Additional File 1: Table S2; Additional File 2: Fig. S5a-b). However, we observed a clear separation between tissues defined by ancestry (Additional File 2: Fig. S5c). No clear separation was observed between different Southern Bantu ethnolinguistic groups, justifying the combination of these samples to reflect a single African ancestral identifier (Additional File 2: Fig. S5d).

**Figure 1:**
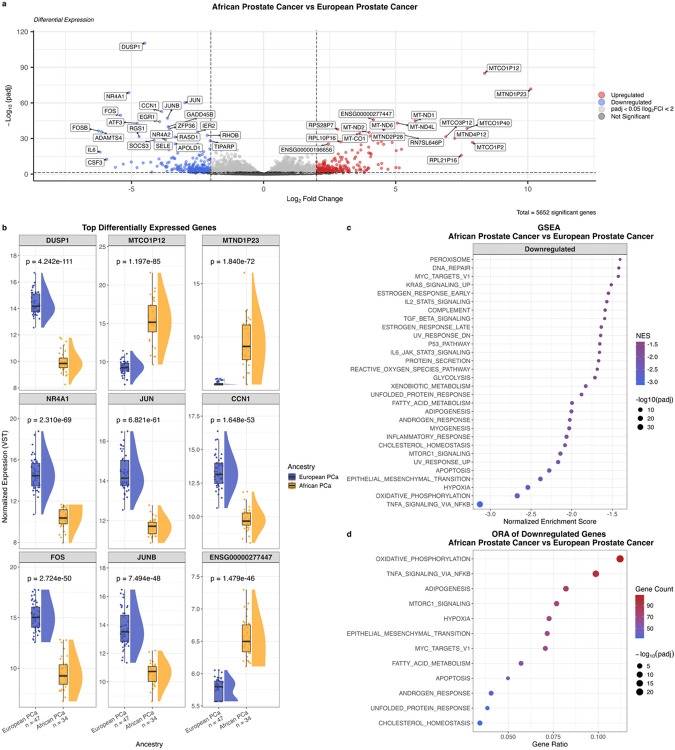
Differential gene expression and pathway analysis between African (n = 34) and European (n = 47) derived GG1 prostate tumours. **a**, Volcano plot of differential gene expression analysis. Genes significantly upregulated (log_2_ fold change > 2, adjusted p < 0.05) in African-derived tumours are shown in red; downregulated genes (log_2_ fold change < 2, adjusted p < 0.05) are shown in blue. Genes with adjusted p < 0.05 but absolute log_2_ fold change < 2 are shown in light grey; non-significant genes are shown in dark grey. **b**, Expression of top differentially expressed genes. Colours indicate ancestry group. P-values from DESeq2 differential expression analysis, Benjamini-Hochberg adjusted. **c**, Gene set enrichment analysis showing significantly enriched pathways (adjusted p < 0.05). Dot colour indicates normalised enrichment score (NES); dot size indicates adjusted p-value. **d**, Overrepresentation analysis of genes significantly downregulated in African-derived tumours (adjusted p < 0.05, log_2_ fold change < 0). Dot colour indicates number of downregulated genes per pathway; dot size indicates adjusted p-value. Gene ratio represents the proportion of downregulated genes found in each pathway. FC = fold change; GSEA = gene set enrichment analysis; NES = normalised enrichment score; ORA = overrepresentation analysis; padj = adjusted p-value; PCa = prostate cancer.

Identifying individual genes driving GG1 tumour transcriptomic differences between the ancestries (34 African vs 47 European), DGE analysis revealed 5,652 genes reaching significance (adjusted *p* < 0.05, Fig. 1a). Among the top-ranked differentially expressed genes (DEGs) by significance (Fig. 1b), six (*DUSP1*, *NR4A1*, *JUN*, *CCN1*, *FOS*, and *JUNB*) were downregulated in African tumours, of which *DUSP1, JUN, FOS*, and *JUNB* are established PCa tumour suppressor genes^41–44^. While *CCN1*/CYR61 has been associated with both tumour promotion and suppression across cancer types^45^, in PCa, higher tumour *CCN1* expression is associated with lower risk of post-surgical recurrence, yet experimental *CCN1* knockdown in PCa advanced, androgen-independent cell lines slows proliferation and reduces TRAIL-induced apoptosis, consistent with a context dependent function^46^. Intriguingly, a recent study showed the human environmental carcinogen, 2,3,7,8-tetrachlorodibenzo-p-dioxin (TCDD) to downregulate *NR4A1* in androgen-dependent PCa cell lines^47^. Conversely, of the three upregulated top-ranked DEGs (*MTCO1P12*, *MTND1P23*, and *ENSG00000277447*) two are mitochondrial pseudogenes, while one is a ribosomal protein pseudogene. *MTND1P23* has been reported to be significantly upregulated in PCa tissue from African American patients compared to that of European American patients^48^.

Gene set enrichment analysis (GSEA) revealed 30 pathways to be differently enriched in African versus European-derived GG1 tumours (adjusted *p* < 0.05, Fig. 1c). Notably, all 30 pathways had a negative normalised enrichment score (NES), indicating downregulation in African tumours. The pathway with the largest absolute NES and the smallest *p*-value was TNFA_SIGNALING_VIA_NFKB, suggesting a less active immune system in African patients. Indeed, six (20.0%) of the 30 pathways were immune-related, with inclusion of INFLAMMATORY_RESPONSE, IL6_JAK_STAT3_SIGNALING, TGF_BETA_SIGNALING, COMPLEMENT and IL2_STAT5_ SIGNALING. Additionally, six (20.0%) of the 30 pathways were metabolism-related, including OXIDATIVE_PHOSPHORYLATION, HYPOXIA, MTORC1_SIGNALING, CHOLESTEROL_ HOMEOSTASIS, FATTY_ACID_METABOLISM, and GLYCOLYSIS, suggesting a different metabolic activity between the ancestries. ORA of the downregulated genes again provided significance for immune and metabolism-related pathways (Fig. 1d). Upregulated DEGs did not show pathway enrichment.

Inspection of the top 20 leading-edge genes in the GSEA (those contributing most to pathway enrichment) validated the biological specificity of the GSEA results. OXIDATIVE_PHOSPHORYLATION showed multiple mitochondrial respiratory chain component genes (Complex I: *NDUFA1, NDUFA2, NDUFA9, NDUFB1, NDUFB2, NDUFB6, NDUFB8, NDUFC1, NDUFS3, NDUFS7*; Complex III: *UQCRQ*; Complex IV: *COX8A*, Complex V: *ATP5ME, ATP5MF, ATP5PD*), confirming pathway-specific downregulation of oxidative phosphorylation in African tumours (Additional File 1: Table S3). Similarly, CHOLESTEROL_HOMEOSTASIS displayed key cholesterol synthesis enzymes (*HMGCR, HMGCS1, IDI1, FDPS, CYP51A1, SQLE, ACAT2, EBP, STARD4*), and FATTY_ACID_METABOLISM showed fatty acid oxidation enzymes (*ACAT2, ACADL, ACSL4*) as leading-edge genes, indicating true metabolic downregulation. In contrast, the GLYCOLYSIS, MTORC1_SIGNALING, and HYPOXIA pathways contained predominantly generic stress response genes rather than pathway-specific genes, suggesting secondary downregulation. For immune pathways, INFLAMMATORY_RESPONSE and TGF_BETA_SIGNALING showed biologically coherent downregulation with pathway-specific leading-edge genes. IL6_JAK_STAT3_SIGNALING, IL2_STAT5_SIGNALING, COMPLEMENT, and TNFA_SIGNALING_VIA_NFKB presented mixed signals with both pathway-specific and generic stress response genes. Collectively, these findings indicate robust downregulation of immune and inflammatory signalling in tumours from African patients.

**Figure 2:**
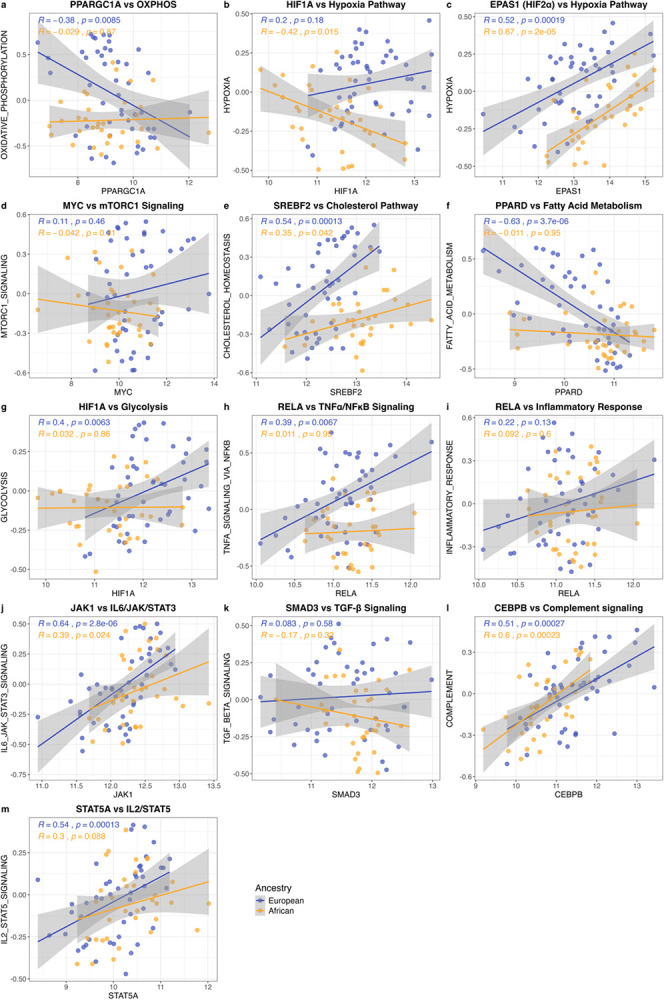
Correlation between transcription factor expression and pathway activity in African (n = 34) and European (n = 47) derived GG1 prostate tumours. **a-m**, Correlation analyses between pathway activity (GSVA scores) and expression of corresponding transcription factors across ancestry groups. Each panel represents one pathway-transcription factor pair, with points coloured by ancestry group. Linear regression lines fitted independently for each group are shown for visualisation. Correlation coefficients (r) and *p*-values calculated using Spearman’s rank correlation. GSVA = gene set variation analysis.

To validate the GSEA findings, we performed GSVA, which confirmed generally lower enrichment scores in African over European tumours for the 12 identified key pathways (Additional File 2: Fig. S6). To explore potential regulatory mechanisms, we examined correlations between key transcription factors (TF) and their pathway GSVA scores^49–57^(Fig. 2a–m; Additional File 2: Fig. S7). While most immune pathway and TF correlations were consistent across ancestry groups (*RELA*-TNF/NFκB, *RELA*-INFLAMMATORY_REPONSE, *JAK1*-IL6_JAK_STAT3, *CEBPB*-COMPLEMENT_SIGNALLING, *STAT5A*-IL2/STAT5A), we observed disparate patterns for HYPOXIA and OXIDATIVE_PHOSPHORYLATION. African-derived tumours showed negative correlation between *HIF1A* expression and HYPOXIA (Spearman r = −0.42, *p* = 0.015), contrary to the no positive trend in European-derived tumours (Spearman r = 0.2, *p* = 0.18). In addition, there was a positive correlation between *HIF2A* expression and HYPOXIA in both ancestry groups (Spearman r = 0.52–0.67, *p* < 0.001). These observations suggest modified regulation of hypoxia-responsive genes in African tumours, although additional studies are required to clarify the functional relevance. In European tumours, *PPARGC1A* and OXIDATIVE_ PHOSPHORYLATION was inversely correlated (Spearman r = −0.38, *p* = 0.0085), which may indicate a compensatory upregulation of *PGC-1α* when oxidative phosphorylation pathway activity is low. This association was not observed for African tumours (Spearman r = 0.029, *p* = 0.87), indicating different regulatory dynamics of oxidative phosphorylation pathway expression. Taken together, our integrated pathway analysis reveals coordinated negative enrichment of several immune and metabolic-related pathways in African-derived GG1-PCa tumours.

**Figure 3:**
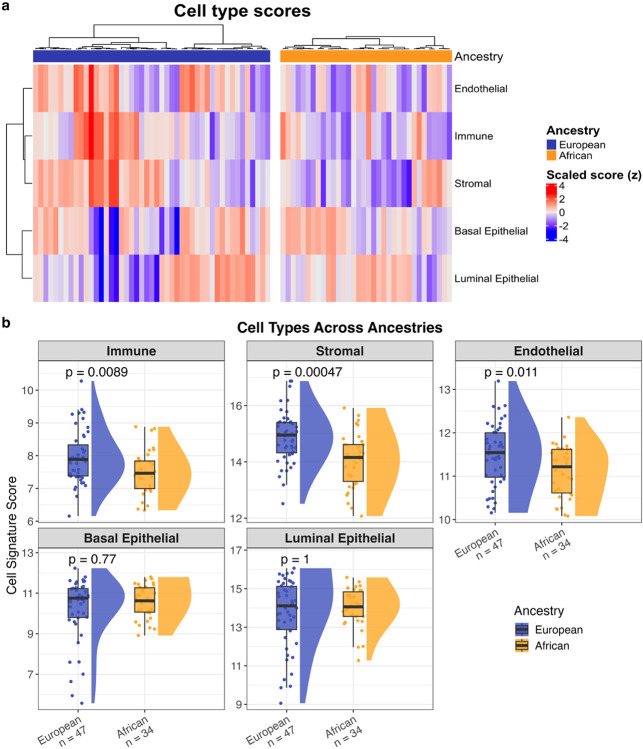
Cell type composition in African (n = 34) and European (n = 47) ancestry GG1 prostate cancer samples. **a**, Heatmap of basal epithelial, luminal epithelial, stromal, endothelial, and immune cell type signature scores. Scores are z-score standardised across samples for visualisation. Samples are clustered within ancestry groups. **b**,Boxplots of cell type scores by ancestry. Colours indicate ancestry group. P-values calculated using Wilcoxon rank-sum test.

To further examine the negative enrichment of immune-related pathways observed for African patients, we analysed the ancestry-specific cellular composition of GG1 tumour tissues. Notably, African tumours showed high luminal and basal epithelial scores similar to European tumours (*p* > 0.05), but lower immune, stromal, and endothelial scores (Fig. 3a–b, Wilcoxon rank-sum test, *p* < 0.05), supporting the downregulation of immune-related pathways. Together, these complementary analyses establish differing transcriptomic landscapes between African and European-derived GG1 prostate tumours.

### Validation of differentially expressed genes in TCGA GG1-PCa data

**Figure 4:**
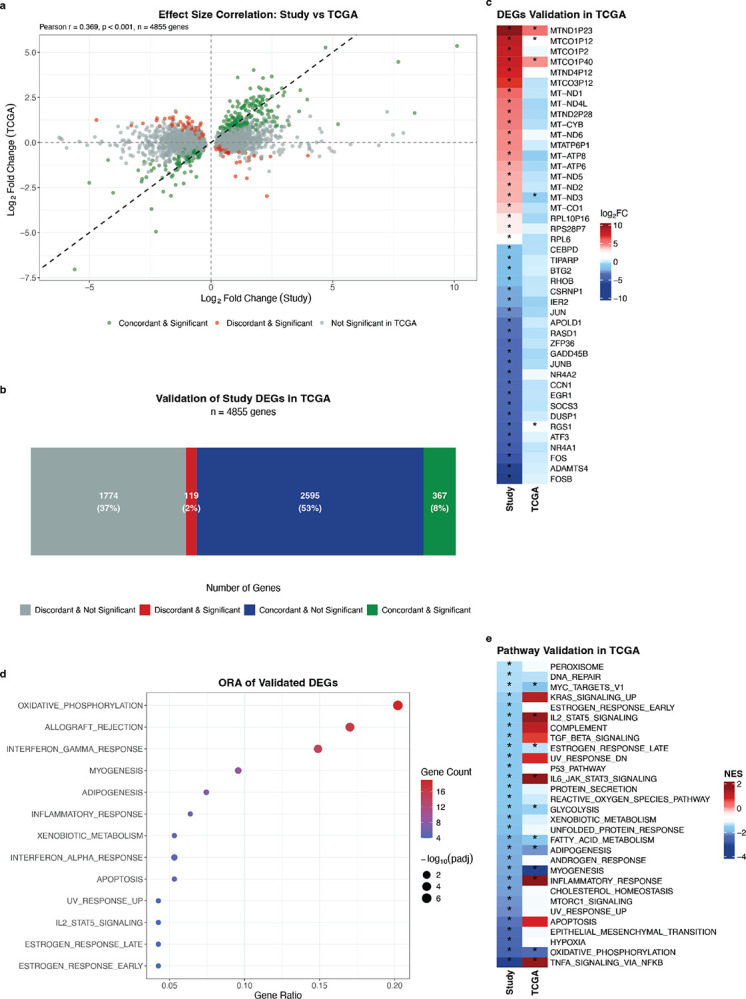
Validation of ancestry-associated differential expression in TCGA GG1-PCa data (13 African American, 48 White American = 48). Analysis restricted to 4,855 genes significantly differentially expressed between African and European ancestry in this study (adjusted p < 0.05) that are also present in TCGA after quality filtering. **a**, Correlation of log_2_ fold changes between this study (South African versus Australian European) and TCGA (African American versus White American). Each point represents one gene, coloured by validation status. Green: significant genes with concordant direction in TCGA (validated); grey: non-significant genes in TCGA; red: significant genes with discordant direction in TCGA. Dashed diagonal line represents perfect concordance. Correlation value calculated using Pearson correlation (r). **b**, Distribution of 4,855 DEGs by validation status in TCGA. Green: significant concordant genes in TCGA; blue: concordant but not significant in TCGA; red: significant discordant genes; grey: non-significant discordant genes. **c**, Heatmap comparing log_2_ fold changes of top 44 DEGs (from top 50 ranked by adjusted p-value in this study, limited to genes present in TCGA) between this study (left) and TCGA (right). Genes ranked by log_2_ fold change in this study. Red indicates upregulation, blue indicates downregulation. Asterisks (*) denote adjusted p < 0.05. **d**, ORA of validated DEGs (n = 367). Only pathways with ≥ 4 genes are shown. Dot colour indicates number of validated DEGs per pathway; dot size indicates adjusted p-value. Gene ratio represents the proportion of validated DEGs found in each pathway. **e**, Comparison of GSEA results showing the 30 pathways significantly enriched in this study (left) and their corresponding enrichment in TCGA (right). Pathways ranked by NES in this study. Asterisks (*) indicate adjusted p < 0.05. DEGs = differentially expressed genes; FC = fold change; GSEA = gene set enrichment analysis; NES = normalised enrichment score; ORA = overrepresentation analysis.

To contextualise our findings within the broader African diaspora, GG1-PCa data was retrieved from TCGA_PRAD, including 13 self-identified Black and 48 White Americans^14^. Representing predominantly western over southern African ancestries, additional cohort differences include earlier age at presentation (median 6.4 years), significantly lower PSA levels (6.1 vs 11.8 ng/mL, *p* = 0.01, Wilcoxon rank-sum test) and lower African over European ancestral representation (Table 1). Of the 5,652 significant DEGs from the African versus European comparison (this study), 4,855 (85.9%) passed QC expression filtering in the TCGA subset, showing modest correlation in fold changes between datasets (Pearson r = 0.369, *p* < 0.001, Fig. 4a). Overall, 367 DEGs (8%) were validated in TCGA with concordant direction of change and statistical significance in both datasets, while an additional 2,595 (53%) showed concordant direction of change without reaching statistical significance (Fig. 4b). Conversely, only 119 (2%) showed significant discordant directions. Detailed examination of the top 50 DEGs in this study, of which 44 were detected in TCGA, revealed three mitochondrial pseudogenes (*MTND1P23*, *MTCO1P40*, and *MTCO1P12*) with directional concordant significance (Fig. 4c). *MTCO1P40* was recently reported to be significantly upregulated in peripheral blood from South African PCa patients compared with USA PCa patients, and in healthy South African controls compared with healthy USA controls^58^. The modest overlap between the datasets suggests ancestry-specific transcriptional differences between PCa in men of west and southern African ancestry, as previously reported for germline PCa susceptibility, including common risk alleles^59^ and rare pathogenic variants^60^.

Further, ORA of the 367 validated genes revealed an overrepresentation of immune-related pathways (5/13 pathways; Fig. 4d), again highlighting the significance of immune-related transcriptional differences in ancestry-related PCa biology. However, contrary to what was observed in this study, in the TCGA subset, immune cell type scores were significantly higher in African Americans compared to White Americans (Additional File 2: Fig. S8). Notably, of the 30 negatively enriched pathways identified in this study, seven (23.3%) were validated as significantly negatively enriched in TCGA (Fig. 4e), indicating greater concordance in pathway-level changes than gene-level changes between the two independent cohorts. Validated pathways included OXIDATIVE_PHOSPHORYLATION, FATTY_ACID_METABOLISM, and GLYCOLYSIS, collectively indicating distinct metabolic pathway activity in African-derived tumours.

### African-specific GG1-PCa gene expression and pathway enrichment

**Figure 5:**
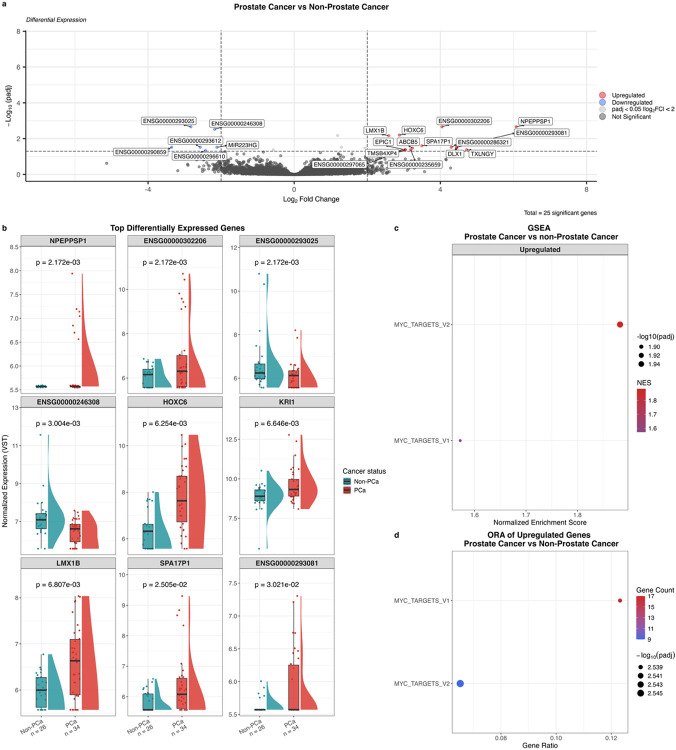
Differential gene expression and pathway analysis between low-grade prostate cancer (PCa, n = 34) and non-PCa (n = 26) tissue from men of African ancestry. **a**, Volcano plot of differential gene expression analysis. Genes significantly upregulated (log_2_ fold change > 2, adjusted p < 0.05) in PCa are shown in red; downregulated genes (log_2_ fold change < 2, adjusted p < 0.05) are shown in blue. Genes with adjusted p < 0.05 but absolute log_2_ fold change < 2 are shown in light grey; non-significant genes are shown in dark grey. **b**, Expression of top differentially expressed genes. Colours indicate cancer status. P-values from DESeq2 differential expression analysis, Benjamini-Hochberg-adjusted. **c**, Gene set enrichment analysis showing significantly enriched pathways (adjusted p < 0.05). Dot colour indicates normalised enrichment score (NES); dot size indicates adjusted p-value. **d**, Overrepresentation analysis of genes nominally significantly upregulated in PCa (unadjusted p < 0.05, log_2_ fold change > 0). Dot colour indicates number of upregulated genes per pathway; dot size indicates adjusted p-value. Gene ratio represents the proportion of upregulated genes found in each pathway. FC = fold change; GSEA = gene set enrichment analysis; NES = normalised enrichment score; ORA = overrepresentation analysis; padj = adjusted p-value; PCa = prostate cancer.

Focusing on our southern African data, we interrogated for DEGs distinguishing GG1-PCa from non-PCa tissues (34 vs 26), identifying 25 of 25,040 genes (adjusted *p* < 0.05, Fig. 5a). Among the top-ranked DEGs by significance, *HOXC6* and *LMX1B* have previously been associated with PCa (Fig. 5b). Consistent with tumour-specific upregulation in our analysis, increased expression has been reported in non-African settings for *HOXC6* and *LMX1B* in PCa compared to normal prostate tissue^37,61^. *NPEPPSP1*, *KRI1*, and *SPA17P1* were significantly upregulated in PCa tissues; however, no prior association with PCa has been reported. Notably, among the top-ranked DEGs, four were uncharacterised long non-coding RNAs (*ENSG00000302206*, *ENSG00000293025*, *ENSG00000246308*, and *ENSG00000293081*), underscoring the gap in genomic annotation in African populations. GSEA showed positive enrichment of MYC_TARGETS_V1 and MYC_TARGETS_V2 in PCa (Fig. 5c). ORA using nominally significantly upregulated DEGs (*p* < 0.05) confirmed MYC pathway enrichment in PCa (Fig. 5d), suggesting MYC pathway activation in African-derived GG1 tumours. Downregulated DEGs did not show pathway enrichment.

### Ancestrally shared GG1-PCa-specific gene expression

**Figure 6:**
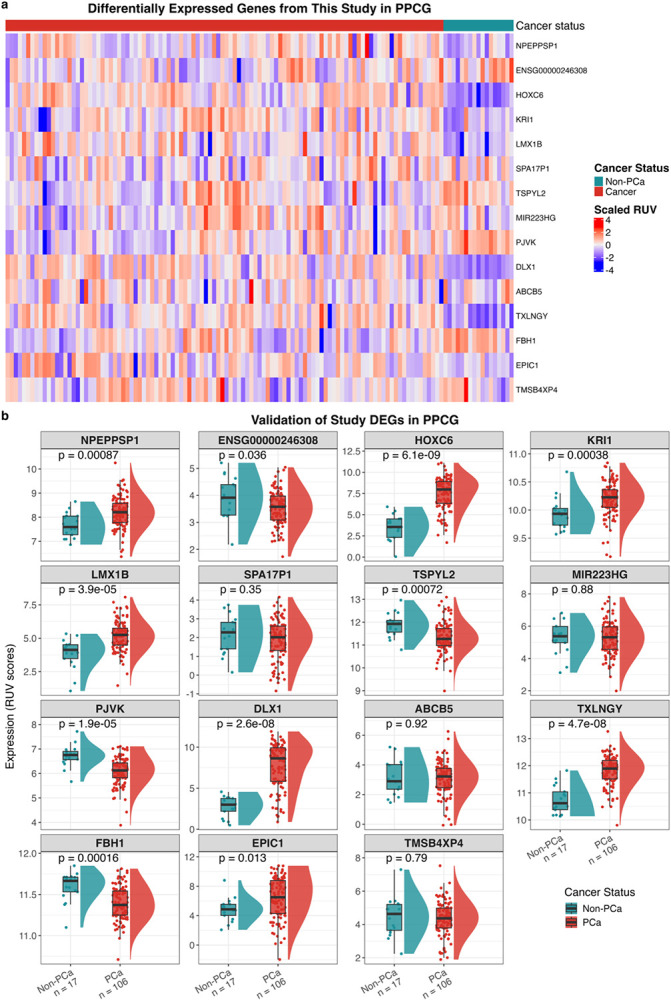
Validation of African-identified GG1 prostate cancer (PCa)-specific differentially expressed genes in an independent non-African cohort (PPCG; PCa = 106, non-PCa = 17). Analysis restricted to 15 genes from the 25 DEGs identified in African PCa versus African non-PCa that are present in PPCG. a, Heatmap of RUV-normalised expression values across PPCG samples. Rows are ordered by adjusted p-value from the study analysis. Expression values are z-score standardised for visualisation. Red indicates high expression; blue indicates low expression. Columns annotated by cancer status. b, Distribution of RUV-normalised expression for each of the 15 genes in PPCG samples. Boxplots ordered by adjusted p-value from the study analysis. Points represent individual samples coloured by cancer status. P-values calculated using Wilcoxon rank-sum test. PCa = prostate cancer.

Lacking non-PCa tissue from our Australian patients, we retrieved total RNA sequencing data from European ancestral GG1-PCa and non-PCa PPCG data (106 vs 17; Table 1). Sourced from Australia (Melbourne), Canada, Denmark, Germany, United Kingdom, and USA, compared with our Australian cohort (Sydney, this study), PPCG GG1 cases presented on average 3.2 years later with PSA levels 1.7-fold higher (*p* = 4.23 × 10^−6^, Wilcoxon rank-sum test). Non-PCa PPCG men, sourced from Denmark, Germany, and the United Kingdom, presented a median of 5 years later with comparable PSA levels to PPCG GG1 cases (7.9 vs 7.4 ng/mL). Of the 25 DEGs in our African GG1-PCa versus non-PCa data, 15 were identified in the non-African dataset, with ten showing significant concordant direction (Fig. 6a–b). Notably, *NPEPPSP1*, *HOXC6, KRI1, LMX1B, DLX1, TXLNGY*, and *EPIC1* were significantly upregulated in GG1-PCa, and *ENSG00000246308*, *PJVK*, and *FBH1* significantly downregulated compared to non-PCa (Fig. 6b). Of these, *HOXC6, LMX1B*, and *DLX1* have been reported to be upregulated in PCa relative to non-PCa in non-African tissues^37,61,62^. Although not reported in PCa, *EPIC1* overexpression is associated with worse prognosis in breast cancer patients and promotes tumour growth through interaction with MYC^63^. The remaining genes (*NPEPPSP1*, *ENSG00000246308*, *KRI1*, *PJVK*, *TXLNGY*, and *FBH1*) lack prior PCa associations. Together, these analyses provide validation of our cancer-specific genes in a non-African cohort.

### PSA-associated transcriptomic differences in African prostate tissue

**Figure 7:**
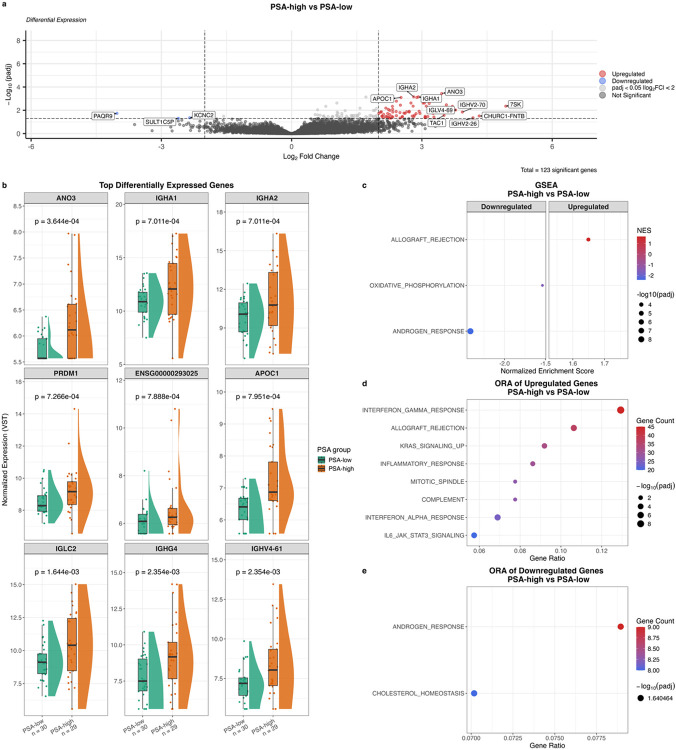
Differential gene expression and pathway analysis between prostate tissues derived from PSA-high (n = 29) versus PSA-low (n = 30) African ancestry participants, irrespective of prostate cancer (PCa) diagnosis **a**, Volcano plot of differential gene expression analysis. Genes significantly upregulated (log_2_ fold change > 2, adjusted p < 0.05) in PSA-high are shown in red; downregulated genes (log_2_ fold change < 2, adjusted p < 0.05) are shown in blue. Genes with adjusted p < 0.05 but absolute log_2_ fold change < 2 are shown in light grey; non-significant genes are shown in dark grey. **b**, Expression of top differentially expressed genes. Colours indicate PSA group. P-values from DESeq2 differential expression analysis, Benjamini-Hochberg-adjusted. **c**, Gene set enrichment analysis showing significantly enriched pathways (adjusted p < 0.05). Dot colour indicates normalised enrichment score (NES); dot size indicates adjusted p-value. **d**, Overrepresentation analysis of nominally significantly upregulated DEGs (unadjusted p < 0.05, log_2_ fold change > 0). Dot colour indicates number of upregulated DEGs per pathway; dot size indicates adjusted p-value. Gene ratio represents the proportion of upregulated DEGs found in each pathway **e**, Overrepresentation analysis of nominally significantly downregulated DEGs (unadjusted p < 0.05, log_2_ fold change < 0). FC = fold change; GSEA = gene set enrichment analysis; NES = normalised enrichment score; ORA = overrepresentation analysis; padj = adjusted p-value; PCa = prostate cancer; PSA = prostate-specific antigen.

Given the modest differences between PCa and non-PCa tissue (25 DEGs) and elevated PSA levels in our southern African men without detectable cancer, irrespective of PCa status, we compared PSA-high and PSA-low tissues (> vs ≤ median 13.75 ng/mL; 29 vs 30) to explore potential undetected disease. DGE analysis revealed 123 DEGs (adjusted *p* < 0.05), of which the vast majority (95.1%) were upregulated in PSA-high tissues (Fig. 7a). Among the top-ranked DEGs by significance (Fig. 7b), all upregulated in PSA-high, *APOC1* is known to be upregulated in PCa compared to normal prostate tissue and promotes apoptosis resistance in PCa cell lines^64^. Although multiple biopsy cores were sampled and pathologically re-reviewed (M.L.), with the sequenced core used entirely for RNA extraction and therefore not graded, these results support our hypothesis that PSA-high tissues may harbour occult PCa. Among the top-ranked DEGs, *ANO3* and *ENSG00000293025* lack prior reports linking them to high PSA levels or PCa. Notably, multiple immune-related genes were among the top-ranked DEGs between PSA-high versus PSA-low tissues (*IGHA1*, *IGHA2*, *IGHG4*, *IGHV4–61*, *IGLC2*, and *PRDM1*), with dual *BATF*/*PRDM1* inhibition in Tregs suppressing tumour growth and metastasis in PCa cell and mouse models^65^. Concordantly, GSEA (Fig. 7c) and ORA (Fig. 7d) revealed positive enrichment of immune-related pathways in PSA-high tissues, while ANDROGEN_RESPONSE, OXIDATIVE_PHOSPHORYLATION, and CHOLESTEROL_HOMEOSTASIS were negatively enriched (Fig. 7c and e), suggesting that PSA elevation in these tissues may occur through AR-independent mechanisms. Interestingly, there was no significant difference in the number of prostatitis cases between the PSA-defined tissue groups (Additional File 2: Fig. S4f). This supports a previous study that found no difference in the presence of prostatitis between controls with PSA levels < 20 and ≥ 20 ng/mL in southern African men^9^.

**Figure 8:**
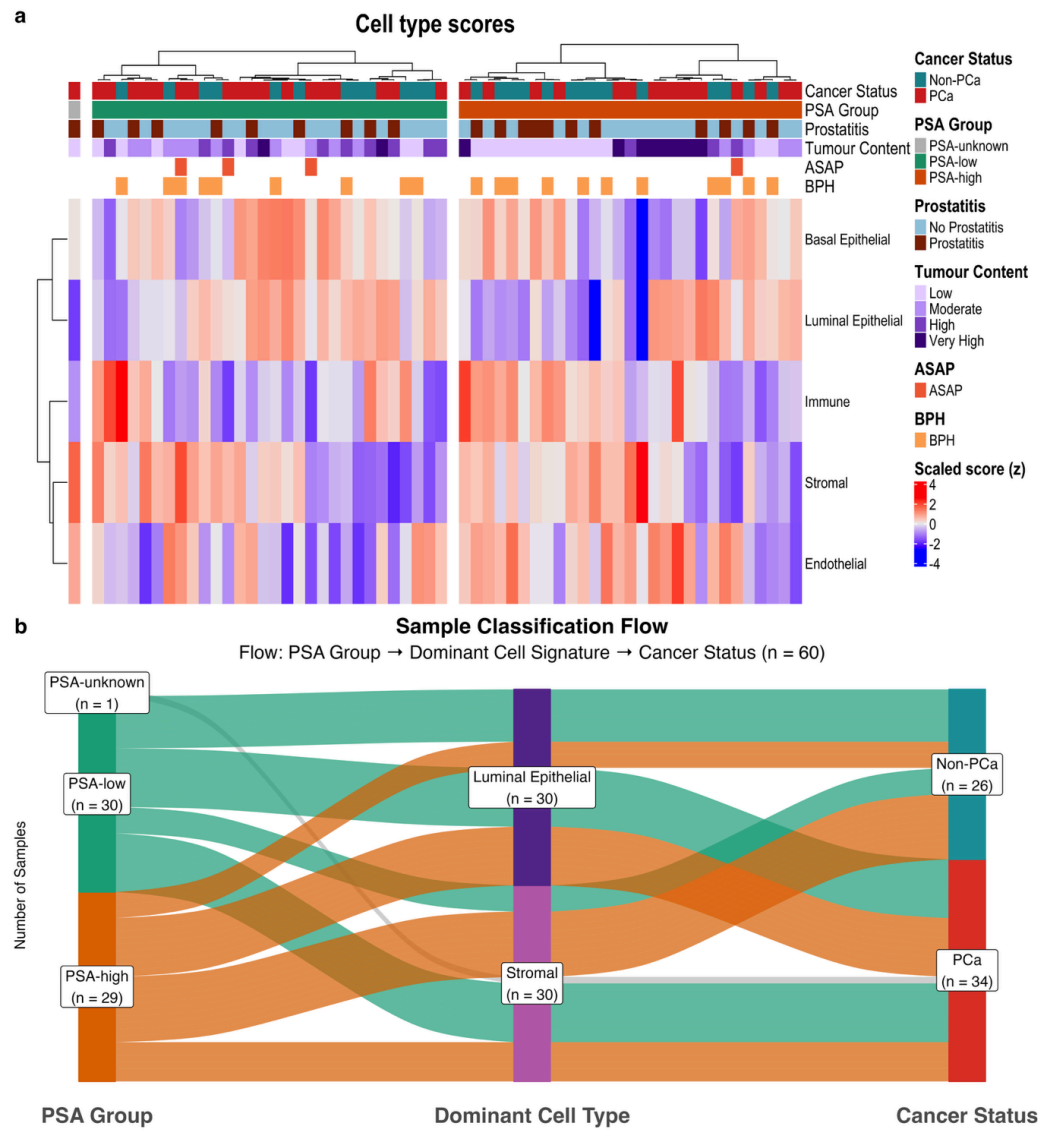
Cell type composition in African-derived PSA-high (n = 29), PSA-low (n = 30) and PSA-unknown (n = 1) tissues. **a**, Heatmap of basal epithelial, luminal epithelial, stromal, endothelial, and immune cell type signature scores. Scores z-score standardised for visualisation, with red indicating high scores and blue indicating low scores. Samples clustered within PSA groups and annotated by clinical and pathological variables. **b**, Sankey diagram showing relationships between PSA levels (left), dominant cell type (middle), and cancer status (right) across 60 samples. Flow width represents sample count; flow colours indicate PSA group. ASAP = Atypical small acinar proliferation; BPH = benign prostatic hyperplasia; PCa = prostate cancer; PSA = prostate-specific antigen.

To investigate cellular composition in tissues from patients with varying PSA levels, we analysed cell type scores in PSA-high versus PSA-low tissues (Fig. 8a; Additional File: Fig. S9). Using hierarchical clustering, two distinct clusters emerged within the PSA-high group. Cluster 1 (n = 16) characterised by high endothelial, stromal, and immune scores and low luminal epithelial scores, comprised predominantly non-PCa tissues (10, 62.5%). Cluster 2 (n = 13), characterised by low stromal and immune scores and high luminal epithelial scores, consisted of predominantly PCa patients (9, 69.2%). Of the four non-PCa tissues in Cluster 2, three had high or moderate estimated tumour content, while one had low tumour content estimation, as observed for the majority of non-PCa samples in the PSA-high group (10/14, 71.4%). Furthermore, Cluster 2 tissues were 1.4-fold less likely to have a recorded incidence of prostatitis. Among the top 25 DEGs, *PIK3CB* and *OPRK1* showed significant upregulation in Cluster 2 versus Cluster 1 (*p* < 4.7^−4^; Additional File 1: Table S4), consistent with reported upregulation in PCa^66,67^.

Flow analysis revealed distinct patterns between PSA-rank, dominant cell type, and disease status (Fig. 8b). Among PSA-high samples (n = 29), most (n = 10) showed stromal cell dominance without PCa, of which seven (70.0%) had diagnosed BPH, consistent with stromal proliferation in this benign condition, and three had chronic prostatitis. Six PSA-high samples exhibited stromal cell dominance with PCa, an unexpected pattern given the epithelial origin of PCa, potentially reflecting tissue heterogeneity or borderline enrichment scores. Four PSA-high samples were luminal epithelial dominant without PCa, potentially reflecting occult disease. Nine PSA-high samples exhibited luminal epithelial dominance with PCa, the classical presentation aligning with the epithelial origin and PSA-producing capacity of malignant luminal cells. Among PSA-low samples (n = 30), four showed stromal dominance without detectable PCa. All had BPH, with one also having prostatitis. Nine exhibited stromal dominance with PCa, including one with ASAP, three with prostatitis, and one with both ASAP and BPH; a similar unexpected pattern potentially reflecting tissue heterogeneity. Eight were luminal-dominant without PCa, of which four had BPH, suggesting early-stage BPH with limited stromal proliferation or glandular-predominant BPH variants. Nine PSA-low tissues showed luminal epithelial dominance with PCa. These patterns indicate that PSA elevation can arise from multiple, biologically distinct mechanisms, including epithelial malignancy, stromal proliferation associated with BPH, and inflammatory processes. Moreover, the cellular composition of prostatic tissue exhibits substantial heterogeneity, even among PCa tissues.

Collectively, these multi-level analyses suggest that transcriptional profiling can identify non-PCa samples with cancer-like characteristics that may represent undetected malignancy. This observation was recently supported through differential methylation profiling for a population-matched cohort of southern African men either with or without clinically confirmed PCa^68^.

## Discussion

**Figure 9:**
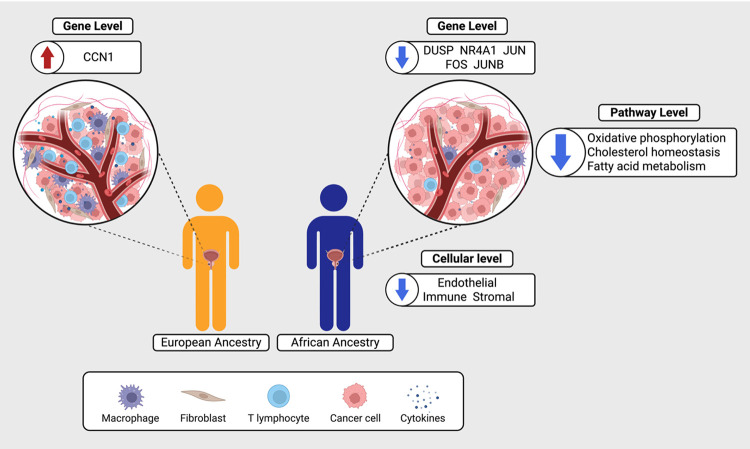
Proposed model of ancestry-associated transcriptional differences in GG1 prostate tumour microenvironment. Schematic illustrating key findings from this study comparing African and European ancestry prostate cancer. African-derived tumours show reduced immune and stromal infiltration, epithelial dominance, impaired cytokine signalling, and metabolic pathway downregulation. Red arrows indicate upregulation; blue arrows indicate downregulation. Top differentially expressed genes with prostate cancer relevance are shown. Created in BioRender. Ferlev Jensby, E. (2026) https://BioRender.com/5el3n6t

As personalised medicine increasingly shapes PCa research and clinical decision-making, the absence of African ancestry patients in RNA sequencing studies leaves a critical gap in knowledge. This is further exasperated by a lack of focus on GG1 disease, where trajectory to lethality is doubled for Black over White American men^4^. Here, generating total RNA sequencing data of prostate tissue from a unique cohort including men from sub-Saharan Africa and a focus on GG1 disease, we characterise gene expression differences between African and European ancestries. We report substantial, multi-level transcriptional divergence in African-derived tumours, including gene expression, pathway enrichment and cell type composition, with suggestive evidence for a potential environmental carcinogenic agent at play (Fig. 9). These findings highlight that current risk stratification tools, developed in European populations, may not adequately capture ancestry-specific molecular features possibly influencing disease progression.

Identifying 5,652 DEGs between African and European-derived GG1-PCa tumours, among the top DEGs by significance, five downregulated genes in African tumours have established roles in PCa, including tumour suppressors *DUSP1*, *JUN, FOS*, and *JUNB*. *DUSP1* inactivation is proposed as an early tumorigenic event^41^, *JUN* regulates senescence and inflammation^42^, *FOS* knockdown induces an oncogenic phenotype in benign prostate cells^43^, and loss of *JUNB* increases proliferation while decreasing senescence^44^. *CCN1* exhibits opposing roles, enhancing TRAIL-induced apoptosis while its knockdown inhibits proliferation^46^. Downregulation of several tumour suppressors may indicate that African-derived low-grade tumours harbour distinct molecular features that are not fully reflected by histological grading systems developed in European populations. Whether these molecular differences translate into different clinical outcomes requires validation in prospective cohorts. Intriguingly, the 4th most significant DEG, *NR4A1*, downregulated in African tumours, is known to be impacted by TCDD^47^. While dioxin levels are reportedly low in Australia, this is not the case in South Africa^69^, including the heavily industrialised Gauteng province^70^, from which 97.1% (33/34) of our cases originate. While all non-PCa participants are from Gauteng, *NR4A1* expression was marginally lower in our PCa tissues (median 10.4 vs 10.3, *p* = 0.5; data not shown). Studies directly correlating expression levels in prostate tissue with measures of TCDD exposure are required to build on a dioxin carcinogenic hypothesis. Of the 4,855 DEGs present in TCGA, 367 (8%) showed significant concordant directional changes in African American versus White American comparisons. The limited overlap emphasises that African ancestry is not monolithic and that findings from African American cohorts are not necessarily transferable across Sub-Saharan Africa. This divergence is further supported by previous studies reporting a 2.1-fold increased risk for advanced PCa in Southern African compared to African American men^6^. Having reported Southern African-specific PCa risk alleles^59^ and pathogenic variants^60,71^, prostate tumour genome-derived cancer drivers and taxonomies^10^ and differential epigenomic regulation^68^ in high-risk disease, our findings highlight the critical need for PCa transcriptomic research conducted in diverse African populations, while further emphasising the need to differentiate GG1 disease.

In this study, 30 pathways were negatively enriched in African tumours, of which 20.0% were immune-related and 20.0% metabolism-related. Although arguably limited by African ancestral differences and lack of study power, it is notable that of the 30 pathways, 23.3% showed significant negative enrichment in African American-derived tumours, with 10% related to metabolism pathways. Notably, Rayford et al., also reported glycolysis downregulation in African American versus European American PCa tissues^48^, supporting the reproducibility of metabolic pathway differences across African-derived populations despite differences in admixture and study design. This suggests that alterations at the pathway-level may be more consistent across studies than individual gene expression changes.

The coordinated downregulation of several immune pathways spanning different mechanisms, including inflammation, cytokine, and complement, suggests broad immune suppression in the African-derived tumour tissues. However, this study cannot determine whether this represents a consequence of the tumour itself or ancestral or environmental factors predisposing tumour development. Nevertheless, the coordinated pattern of downregulation across functionally distinct immune pathways suggests ancestry-associated biological differences in immune pathway regulation that warrant further investigation. Concordantly, cell type analysis revealed significantly lower immune, stromal and endothelial scores in African-derived tumours. PCa is generally characterised as having an immunologically cold TME^34^. Our findings suggest greater immune suppression in African-derived tumours, which has been associated with worse clinical outcomes^72^. This is consistent with previously reported upregulation of serum protein signatures indicative of tumour immune suppression in PCa patients of African ancestry, which correlates with metastatic and lethal disease^73^, and with virtually no cytotoxic T lymphocyte or NK cell infiltration in the tumour area of Black sub-Saharan African men with PCa^74^. The epithelial-dominant TME in African-derived tumours indicates that tumour development is primarily driven by epithelial cell-intrinsic mechanisms rather than immune or stromal signals. Consistent with this, positive MYC pathway enrichment in African-derived PCa versus non-PCa tissue reflects epithelial proliferation. In contrast, in TCGA, immune scores were significantly higher in African American versus White American patients. The TCGA subset comprised only 13 African American individuals of admixed ancestry, compared to the predominantly non-admixed South African population in this study. This discrepancy in ancestry composition and small TCGA sample size may underlie the observed differences in immune scores. Notably, Rayford et al. also reported higher immune pathway activity in African American versus European American PCa tumours^48^. This discordance may, beside ancestry composition differences, reflect differences in RNA profiling platforms (microarray gene expression profiling versus total RNA sequencing), or biological heterogeneity within African-derived populations. These conflicting findings raise questions about whether such transcriptional differences might influence immunotherapy response and emphasise the need for larger studies in diverse African populations.

In contrast to immune pathways, which generally showed expected positive correlation between TF and pathway activity in both ancestry groups, metabolic TF and pathway correlations showed less consistency, suggesting post-transcriptional alteration beyond plain transcriptional control. PCa typically maintains oxidative metabolism, increases fatty acid metabolism and cholesterol biosynthesis^75^; however, these pathways showed pathway-specific downregulation in African-derived prostate tissues in our study. As such, we hypothesise that African-derived tumours may constitute a metabolically distinct subtype within low-grade PCa, potentially relying on alternative metabolic pathways for energy production. Although glycolysis exhibited negative enrichment in African-derived tumours relative to European-derived, this signal was largely driven by generic stress-response genes. Consequently, it remains plausible that glycolysis serves as an important energy source for malignant cells in African prostate tumours. An alternative explanation is that differences in metabolic efficiency could lead to an apparent negative enrichment of these pathways. These hypotheses warrant investigation in future studies. Taken together, our findings reveal that low-grade PCa encompasses ancestry-specific transcriptomic heterogeneity, which may result in current risk classification tools missing clinically relevant biology in African ancestry patients.

Beyond these ancestry-specific features, we identified cancer-specific transcriptional changes within the African cohort. Revealing only 25 significant DEGs, 40% showed significant concordant direction in the non-African-derived low-grade PCa versus non-PCa matched PPCG dataset, providing cross-ancestry validation. Notably limited by sample sizes, the modest amount of DEGs between African PCa and non-PCa may reflect occult PCa in the non-PCa group, particularly given that South African patients were diagnosed through TRUS, which have lower sensitivity compared to magnetic resonance imaging (MRI)-guided biopsies^16^, increasing the risk of missed diagnoses. This is consistent with our PCA, which showed no clear separation between PCa and non-PCa samples. Interestingly, in a recently published study, using differential methylation analysis for regionally matched prostate tissues, we reported 10.8% (7/65) non-PCa southern African-derived tissues to exhibit tumour-like methylation profiles^68^. Intriguingly, it was evident in this study that the transcriptional differences between PCa and non-PCa were dwarfed by the ancestry-related differences, with an above 200-fold difference in DEGs (5,652 vs 25). For this reason, it was notable that when comparing PSA-high versus PSA-low within the African tissues, irrespective of PCa status, we found a 4.9-fold higher number of DEGs over PCa versus non-PCa. Cell type analysis further revealed four PSA-high, non-PCa samples that displayed PCa-like luminal epithelial profiles, supporting the hypothesis of occult disease. These findings further emphasise the need to adopt the more sensitive MRI-guided approaches^16^, particularly for anteriorly placed tumours, which are common in African-ancestry men^17^. Additionally, our results highlight the poor diagnostic performance of PSA in this population^76^ and underline the need for ancestry-specific biomarkers.

Some limitations warrant consideration. Although limited by study size, this is arguably the largest and only study of its kind for sub-Saharan Africa, while studies focused on low-grade PCa of any population are scarce. The latter, perpetuated by the predominance of aggressive disease presentation across the continent due to limited screening access and delayed detection^6,77^. In turn, providing African-relevant validation is currently limited both by study size (13 TCGA-derived tissues) and ancestral fractions, southern versus western African. While southern Africans represent the tip of Bantu migration from a roughly 5,000-year-old west Bantu origin^78^, lacking non-African admixture, southern Bantu populations carry a proportionate early-diverged and genetically rich Khoe-San contribution^79^. Conversely, the 17th -century transatlantic slave-trade brought predominantly West African heritage (71.3% ± 22%) to modern-day African Americans, with on average 28.7% non-African admixture^80^. Again, we have demonstrated that ancestry differences between our southern African and African American populations are interpretable as genetic differences in both PCa-associated risk alleles, including polygenic risk scores, and pathogenic variants^59,60,71^. Furthermore, as a subset of southern African non-PCa tissues show both transcriptional (this study) and methylation^68^ tumour-like profiles, we speculate that a subset of these non-PCa patients have a misdiagnosis, while absence of European-derived non-PCa tissues precluded direct comparison using our single study design and workflow. While we speculate on a potential link between TCDD exposure and differential expression in South Africa, our study cannot disentangle genetic influences from environmental factors in driving the ancestry-associated differences. To address this gap, exposomics data are currently being collected and analysed as part of the Southern African Prostate Cancer Study (SAPCS) and Health Equity Research Outcomes and Improvement Consortium Prostate Cancer Precision Health Africa1K (HEROIC PCaPH Africa1K)^81^. Lastly, the lack of follow-up data precludes assessment of whether the transcriptional differences observed between ancestries associate with clinical outcomes such as progression or metastasis.

To our knowledge, no previous study has performed comprehensive transcriptomic profiling of prostate tumours from sub-Saharan African men, and the limited existing molecular analyses have focused predominantly on high-grade disease. Here, we performed total RNA sequencing of GG1 tumours from African and European ancestry men, revealing distinct transcriptomic profiles. African-derived tumours exhibited substantial downregulation of multiple tumour suppressor genes and metabolism and immune-related pathways, with significantly lower immune, stromal, and endothelial cell type scores. These transcriptomic differences raise questions about whether current risk stratification tools, largely developed in European populations, adequately capture ancestry-specific molecular features influencing disease progression. Prospective studies with clinical outcomes are needed to determine whether these transcriptional patterns are associated with differences in disease behaviour.

## Supplementary Material

Supplementary Files

This is a list of supplementary files associated with this preprint. Click to download.

- AdditionalFile1SupplementaryTables.pdf
- AdditionalFile2SupplementaryFigures.pdf

Source data

Excel files containing the source data used to generate the main figures of this study are provided as supplementary materials.

## Figures and Tables

**Table 1:** Participant clinicopathological characteristics for this study (n = 116), with comparative data from The Cancer Genome Atlas (TCGA, n = 61) and Pan Prostate Cancer Group (PPCG, n = 123).

Characteristic	This study			TCGA		PPCG	
	African		European	African	European	European	
	Non-PCa n = 28	PCa n = 40	PCa n = 48	PCa n = 13	PCa n = 48	Non-PCa n = 17	PCa n = 106
**Country**^a^, **n (%)**							
South Africa	28 (100)	40 (100)	0 (0)	0 (0)	0 (0)	0 (0)	0 (0)
Australia	0 (0)	0 (0)	48 (100)	0 (0)	1 (2.1)	0 (0)	3 (2.8)
USA	0 (0)	0 (0)	0 (0)	13 (100)	40 (83.3)	0 (0)	29 (27.4)
Other	0 (0)	0 (0)	0 (0)	0 (0)	7 (14.6)	17 (100)	74 (69.8)
**Age in years, median (IQR)** ^b^	64.5 (58.0–66.2)	64.0 (60.8–69.0)	56.8 (53.0–60.1)	57.6 (56.3–60.7)	58.1 (53.5–62.4)	65.0 (61.0–70.0)	60.0 (50.2–66.0)
**PSA ng/mL, median (IQR)** ^c^	14.4 (8.2–19.4)	11.8 (8.2–41.1)	4.4 (3.4–5.7)	6.1 (4.3–8.7)	6.1 (4.5–7.0)	7.9 (5.2–11.2	7.4 (5.6–10.7)
**BPH**, **n (%)**	20 (71.4)	0 (0)	NA	NA	NA	NA	NA
**Prostatitis**, **n (%)**	14 (50.0)	9 (22.5)	NA	NA	NA	NA	NA
**ASAP**, **n (%)**	0	6 (15.0)	NA	NA	NA	NA	NA
**Grade Group**^d^, **n (%)**							
1	NA	34 (85.0)	48 (100)	13 (100)	48 (100)	NA	106 (100)
NA	28 (100)	0 (0)	0 (0)	0 (0)	0 (0)	17 (100)	0 (0)

a Country of birth and recruitment for this study, country of residence at enrolment for TCGA, and country of recruitment for PPCG, with other referring to Canada, Denmark, Germany, and the UK.

b Age at diagnosis in this study and TCGA, and at tumour collection for PPCG. Age unavailable for two African American and one White American TCGA patients, and one non-PCa and 36 PCa PPCG patients.

c PSA at diagnosis for this study, PSA before radical prostatectomy in TCGA, PSA at tumour collection for PPCG. PSA unavailable for one African and six European PCa patients in this study, and five African American and 21 White American TCGA patients, and one non-PCa and 42 PCa PPCG patients.

d Grade group from diagnostic biopsies for African ancestry and radical prostatectomy for European ancestry patients in this study. Grade Group from radical prostatectomy for TCGA and PPCG. ASAP = atypical small acinar proliferation; BPH = benign prostatic hyperplasia; IQR = interquartile range; NA = not available; PCa = prostate cancer; PSA = prostate-specific antigen.

## Data Availability

Total RNA-sequencing data generated in this study are available via the European Genome-Phenome Archive (EGA) [https://ega-archive.org] under study accession [XXXXX available at publication]. Access requires Data Access Committee (DAC) approval in line with project-specific access policies. The cohort comprises prostate tissue samples from South African men (n = 68, PCa = 40, non-PCa = 28) and Australian men with PCa (n = 48).

## Code availability

Source code for analysis and figure production is available on GitHub (https://github.com/Efjensby/african-low-grade-prostate-rnaseq).

## Southern African Prostate Cancer Study (SAPCS)

**Leads**: M.S. Riana Bornman^1,2^, Shingai B.A. Mutambirwa^3^, Vanessa M. Hayes^1,4,5,6^; **Key Members (alphabetical order)**: Oseghau O. Aire^7,8^, Raymond A. Campbell^7,8^, Maphuti Tebogo Lebelo^9^, Melanie Louw^10,11^, Tumisang M.N. Mbeki^1^, Reginold M. J. Menoe^12^, Lekhotla Richard Monare^13^, M. Viola Morolo^7^, Mukudeni Nenzhelele^14^, Muvhulawa Obida^1,14^, Martin Obida^14^, Sean M. Patrick^1^, Mulalo B. Radzuma^3^, Joyce Shirinde^1^, Smit van Zyl^13^

^1^School of Health Systems and Public Health, Faculty of Health Sciences, University of Pretoria, Pretoria, Gauteng, South Africa; ^2^Department of Biological Sciences, Faculty of Science, Engineering and Agriculture, University of Venda, Limpopo, South Africa; ^3^Department of Urology, Sefako Makgatho Health Science University, Dr George Mukhari Academic Hospital, Ga-Rankuwa, Gauteng, South Africa; ^4^Ancestry and Health Genomics Laboratory, Charles Perkins Centre, School of Medical Sciences, Faculty of Medicine and Health, University of Sydney, Camperdown, NSW 2050, Australia; ^5^Manchester Cancer Research Centre, University of Manchester, Manchester, UK; ^6^Norwich Medical School, University of East Anglia, Norwich, UK; ^7^Department of Urology, University of Pretoria, Steve Biko Academic Hospital, Pretoria, Gauteng, South Africa; ^8^Department of Urology, Kalafong Academic Hospital, Pretoria, Gauteng, South Africa; ^9^Department of Physiology, Faculty of Health Sciences, University of Pretoria, Pretoria, South Africa; ^10^National Health Laboratory Services, Johannesburg, South Africa; ^11^Department of Anatomical Pathology, School of Pathology, University of the Witwatersrand, Johannesburg, South Africa; ^12^Life Peglerae Hospital, Rustenberg, North West, South Africa; ^13^Polokwane Urology, Limpopo, South Africa; ^14^SAPCS Unit, Tshilidzini Hospital, Shayandima, Thohoyandou, Limpopo, South Africa

## Pan Prostate Cancer Group (PPCG)

**Leads**. Rosalind A. Eeles^1,2^, Colin S. Cooper^1,3^; **RNA Working Group Leads**: Anthony T. Papenfuss^4,5^, Luigi Marchionni^6^, **Key Members (alphabetical order)**: G. Steven S. Bova^7^, Daniel S. Brewer^3,8^, Robert G. Bristow^9^, Mark N. Brook^1^, Benedict Brors^10,11^, Adam Butler^12^, Geraldine Cancel-Tassin^13,14^, Kevin C. L. Cheng^15,16^, Niall M. Corcoran^17,18,19^, Olivier Cussenot^13,14^, Francesco Favero^20,21^, Clarissa Gerhauser^10^, Abraham Gihawi^3^, Etsehiwot G. Girma^20,21^, Vincent J. Gnanapragasam^22^, Andreas J. Gruber^23^, Anis Hamid^19^, Vanessa M. Hayes^3,9,24,25^, Housheng Hansen He^26^, Chris M. Hovens^17,18,19^, Eddie Luidy Imada^6^, G. Maria Jakobsdottir^9,27^, Chol-Hee Jung^28^, Francesca Khani^6^, Zsofia Kote-Jarai^1^, Philippe Lamy^29,30^, Gregory Leeman^10^, Massimo Loda^6^, Pavlo Lutsik^10,31^, Ramyar Molania^4,5^, Diogo Pellegrina^15^, Bernard Pope^28,32^, Lucio R. Queiroz^6^, Tobias Rausch^33^, Jüri Reimand^15,16^, Brain Robinson^6^, Atef Sahli^9^, Thorsten Schlomm^34^, Karina Dalsgaard Sørensen^29,30^, Sebastian Uhrig^10^, David C. Wedge^9^, Joachim Weischenfeldt^34,35^, Yaobo Xu^12^, Takafumi N. Yamaguchi^36^, Claudio Zanettini^6^

^1^The Institute of Cancer Research, London, UK; ^2^The Royal Marsden NHS Foundation Trust London, UK; ^3^Norwich Medical School, University of East Anglia, Norwich, UK; ^4^The Walter and Eliza Hall Institute of Medical Research, Melbourne, Australia; ^5^Department of Medical Biology, The University of Melbourne, Melbourne, Australia; ^6^Department of Pathology and Laboratory Medicine, Weill Cornell Medicine, New York, USA; ^7^Prostate Cancer Research Center, Faculty of Medicine and Health Technology, Tampere University, Tampere, Finland; ^8^The Earlham Institute, Norwich Research Park, Norwich, UK; ^9^Manchester Cancer Research Centre, University of Manchester, Manchester, UK; ^10^German Cancer Research Center (DKFZ), Heidelberg, Germany; ^11^Medical Faculty Heidelberg and Faculty of Biosciences, Heidelberg University, Germany; ^12^Wellcome Sanger Institute, Cambridge, UK; ^13^CeRePP, Hospital Tenon, Paris, France; ^14^Sorbonne Universite, GRC n^o^5 Predictive Onco-Urology, APHP, Tenon Hospital, Paris, France; ^15^Computational Biology Program, Ontario Institute for Cancer Research, Ontario, Canada; ^16^Department of Medical Biophysics, University of Toronto, Toronto, Canada; ^17^Collaborative Center for Genomic Cancer Medicine University of Melbourne, The Victorian Comprehensive Cancer Centre, Parkville, Victoria, Australia; ^18^Department of urology, Royal Melbourne Hospital, Melbourne, Parkville, Victoria, Australia; ^19^Department of Surgery, The University of Melbourne, Parkville, Victoria, Australia; ^20^Biotech Research and Innovation Centre, University of Copenhagen, Copenhagen, Denmark; ^21^Finsen Laboratory, Copenhagen University Hospital Rigshospitalet, Copenhagen, Denmark; ^22^Department of Surgery, University of Cambridge and Cambridge University Hospitals NHS Trust, Cambridge Biomedical Campus, Addenbrooke’s Hospital, Cambridge, UK; ^23^University of Konstanz, Konstanz, Germany; ^24^Ancestry and Health Genomics Laboratory, Charles Perkins Centre, School of Medical Sciences, Faculty of Medicine and Health, University of Sydney, Camperdown, NSW 2050, Australia; ^25^School of Health Systems and Public Health, University of Pretoria, Pretoria, South Africa; ^26^Princess Margaret Cancer Centre, University Health Network, Department of Medical Biophysics, University of Toronto, Toronto, Canada; ^27^Christie Hospital, The Christie NHS Foundation Trust, Manchester Academic Health Science Centre, Manchester, UK; ^28^Melbourne Bioinformatics, The University of Melbourne, Melbourne, Australia; ^29^Department of Molecular Medicine, Aarhus University Hospital, Aarhus, Denmark; ^30^Department of Clinical Medicine, Aarhus University, Aarhus, Denmark; ^31^Department of Oncology, KU Leuven, Leuven, Belgium; ^32^Australian BioCommons, The University of Melbourne, Melbourne, Australia; ^33^Genome Biology, European Molecular Biology Laboratory (EMBL), Heidelberg, Germany; ^34^Charité Universitätsmedizin Berlin, Berlin, Germany; ^35^Biotech Research & Innovation Centre & Finsen Laboratory, University of Copenhagen, Rigshospitalet, Copenhagen, Denmark; ^36^Jonsson Comprehensive Cancer Center, University of California Los Angeles, Los Angeles, CA, USA.

## Abbreviations

ASAP: Atypical small acinar proliferation
BPH: Benign prostatic hyperplasia
DEG: Differentially expressed gene
DGE: Differential gene expression
GG: Grade Group
GSEA: Gene set enrichment analysis
GSVA: Gene set variation analysis
HEROIC PCaPH: Health Equity Research Outcomes and Improvement Consortium Prostate Cancer Precision Health
ISUP: International Society of Urological Pathology
IQR: Interquartile range
MRI: Magnetic resonance imaging
NA: Not available
NCCN: National Comprehensive Cancer Network
NES: Normalised enrichment score
ORA: Overrepresentation analysis
PCa: Prostate cancer
PCA: Principal component analysis
PPCG: Pan Prostate Cancer Group
PSA: Prostate-specific antigen
QC: Quality check
RP: Radical prostatectomy
SAPCS: Southern African Prostate Cancer Study
TCDD: Tetrachlorodibenzo-p-dioxin
TCGA: The Cancer Genome Atlas
TF: Transcription Factor
TME: Tumour microenvironment
TRUS: Transrectal ultra-sound guided
